# QSAR and molecular docking for the search of AOX inhibitors: a rational drug discovery approach

**DOI:** 10.1007/s10822-020-00360-8

**Published:** 2020-12-08

**Authors:** Alicia Rosell-Hidalgo, Luke Young, Anthony L. Moore, Taravat Ghafourian

**Affiliations:** 1 Department of Biochemistry and Biomedicine, School of Life Sciences, University of Sussex, Falmer, Brighton, BN1 9QG UK; 2 School of Life Sciences, Faculty of Creative Arts, Technologies and Science, University of Bedfordshire, Luton, Bedfordshire, LU1 3JU UK

**Keywords:** AOX, Ascofuranone, Molecular docking, QSAR, Alternative oxidase, Fungicide

## Abstract

The alternative oxidase (AOX) is a monotopic diiron carboxylate protein that catalyses the oxidation of ubiquinol and the reduction of oxygen to water. Although a number of AOX inhibitors have been discovered, little is still known about the ligand–protein interaction and essential chemical characteristics of compounds required for a potent inhibition. Furthermore, owing to the rapidly growing resistance to existing inhibitors, new compounds with improved potency and pharmacokinetic properties are urgently required. In this study we used two computational approaches, ligand–protein docking and Quantitative Structure–Activity Relationships (QSAR) to investigate binding of AOX inhibitors to the enzyme and the molecular characteristics required for inhibition. Docking studies followed by protein–ligand interaction fingerprint (PLIF) analysis using the AOX enzyme and the mutated analogues revealed the importance of the residues Leu 122, Arg 118 and Thr 219 within the hydrophobic cavity. QSAR analysis, using stepwise regression analysis with experimentally obtained IC_50_ values as the response variable, resulted in a multiple regression model with a good prediction accuracy. The model highlighted the importance of the presence of hydrogen bonding acceptor groups on specific positions of the aromatic ring of ascofuranone derivatives, acidity of the compounds, and a large linker group on the compounds on the inhibitory effect of AOX.

**Electronic supplementary material:**

The online version of this article (10.1007/s10822-020-00360-8) contains supplementary material, which is available to authorized users.

## Introduction

The alternative oxidase (AOX) is a non-protonmotive ubiquinol–oxygen oxidoreductase that couples the oxidation of ubiquinol with the complete reduction of oxygen to water in a manner insensitive to inhibitors of the cytochrome oxidase pathway [1, 2]. In eukaryotes, AOXs are located attached to the inner surface of the inner membrane of the mitochondria, on the substrate-side of the cytochrome *bc*_*1*_ complex [3]. Historically, the AOX was first identified in thermogenic plants, however the gene encoding this protein has been found in all higher plants and also throughout other kingdoms such as the fungal, protist and in prokaryotes [4]. Homologs have also been identified in α-proteobacteria, cyanobacteria and in some animals such as molluscs, nematodes and chordates, but not in mammals [5]. The physiological functions of AOXs vary between organisms, but typically include thermogenesis, stress tolerance and maintenance of mitochondrial and cellular homeostasis [2].

The AOX is widespread among some important human pathogenic parasites such as *Blastocystis hominis*, the most common eukaryotic microbe found in the human gut [6], *Paracoccidioides brasiliensis*, the pathogenic fungus responsible for paracoccidioidomycosis in humans [7], or *Candida albicans*, an opportunistic human pathogen [8]. The AOX is also found in *Cryptosporidium parvum,* one of the most widespread intestinal parasites, responsible for diarrheal disease cryptosporidiosis, for which there is no effective treatment currently available. Cryptosporidiosis represents a potential fatal disease especially in opportunistic infections in immunodeficient individuals [9, 10]. However, amongst all these, the AOX has been most intensely investigated for its crucial role in the energy metabolism of the African trypanosomes [11].

African trypanosomes of the genus *Trypanosoma*, classified as human (*Trypanosoma brucei gambiense* and *Trypanosoma brucei rhodesiense*) or animal (*T. b. brucei, T. congolense* and *T. vivax*) are the etiological agents of human African sleeping sickness or trypanosomiasis (HAT) and nagana in cattle, respectively. They are transmitted to man and animals by the bite of the *Glossina spp.* insect, commonly known as the tsetse fly. African trypanosomiasis is endemic to sub-Saharan Africa—with approximately 70 million people at different levels of risk of contracting HAT, it is considered one of the leading neglected tropical diseases [12]. Without prompt diagnosis and treatment, the disease is usually fatal: the parasites reside in the bloodstream, multiply in the body, cross the blood–brain barrier and invade the central nervous system [12]. Currently, there is no effective vaccine for its prevention and treatments are far from satisfactory due to the toxicity and complex administration of the available drugs. Only a few drugs are registered for the treatment of HAT: pentamidine, suramin, melarsoprol, eflornithine and, only under special authorisation, nifurtimox in combination with eflornithine. However, all these drugs are problematic due to associated severe adverse effects and toxicities, resistance and cross reaction with human targets [13]. For instance, eflortnithine is the only drug with a defined target, the ornithine decarboxylase, and still it has poor potency against *T. brucei *[13]*.* Clearly, new drugs with known mechanisms of action are urgently required.

Given the integral role that the AOX plays in respiration and its increasing occurrence in relevant pathogenic organisms, it has become an important drug target to consider. The trypanosome alternative oxidase (TAO) represents a unique and safe therapeutic target, not only because it is absent from mammals, but also because it plays a critical role in the survival of the parasite in its bloodstream form [14].

**Fig. 1:**
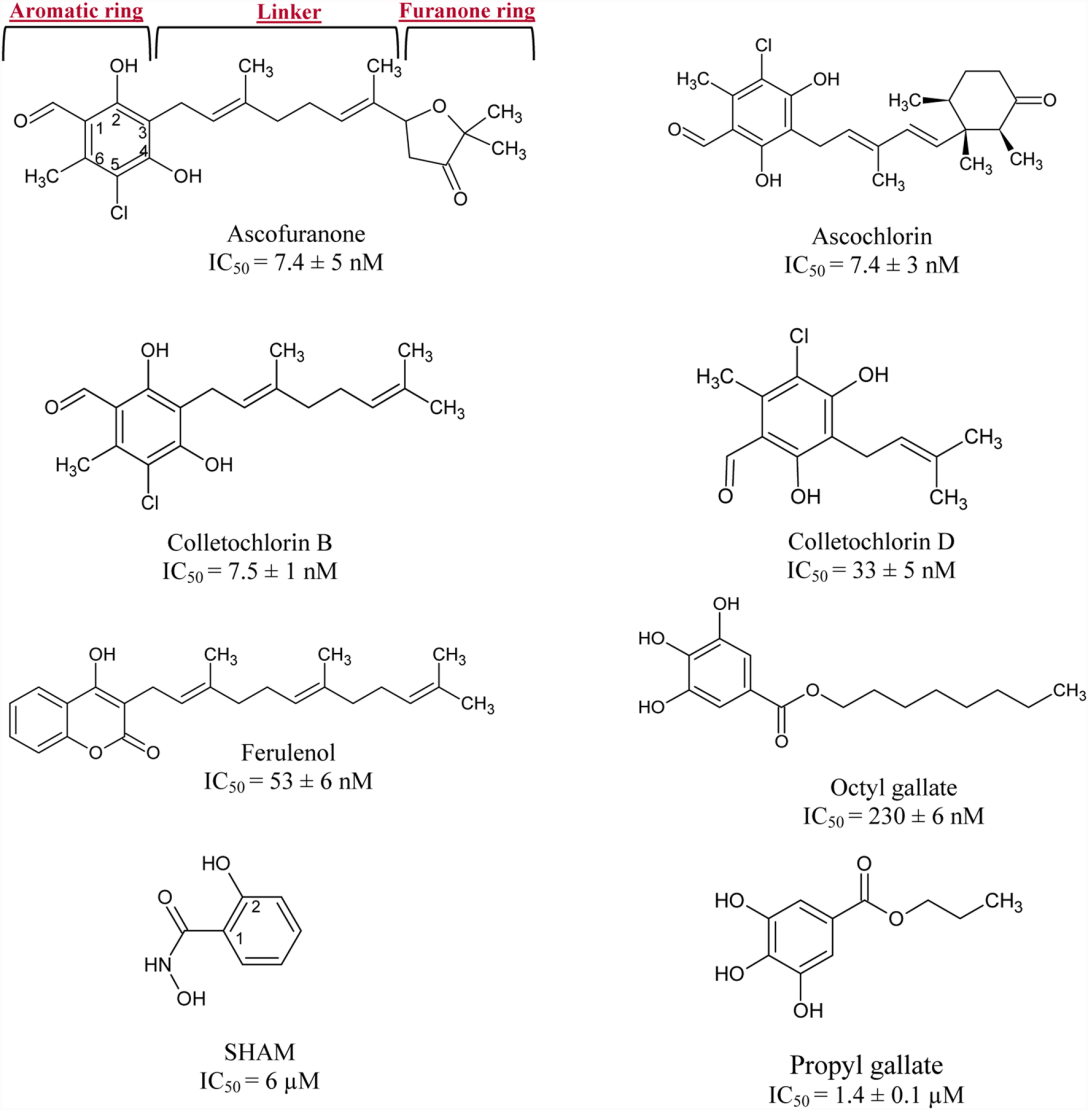
Chemical structure of AOX inhibitors and IC_50_ values obtained for recombinant AOX expressed in *E.* c*oli* membranes. Data are mean ± SD of n = 3 isolations

TAO has been considered a valid target for decades, which has led to the identification of a number of specific inhibitors, the structures of which are summarized in Fig. 1. Among these, SHAM and propyl gallate represent the most studied inhibitors [15, 16]. Ascofuranone (AF), an antibiotic isolated from the pathogenic fungus *Ascochytia visiae,* is the most potent inhibitor of the AOX family enzymes identified to date. It specifically inhibits TAO at subnanomolar concentrations (IC_50_ = 0.13 nM) [17]. In contrast, SHAM (IC_50_ = 4 μM) and propyl gallate (IC_50_ = 200 nM) require higher concentrations for inhibition [17]. AF has been known for over 20 years to have trypanocidal activity both in vitro and in vivo, with successful treatment of the *T. vivax*-infected mouse, at a single intramuscular dose of 50 mg/kg or 6 mg/kg on 4 consecutive days [19, 20]. However, AF is still far from being the perfect candidate for treating HAT due to some unwanted molecular features, which account for its rapid blood clearance, low oral bioavailability and potential toxicity [11]. Even though AF undoubtedly constitutes a promising lead compound, the reality is that we still lack a TAO inhibitor at an advanced stage of clinical development despite TAO’s unique and critical role. Firstly, this could be explained by the fact that the complex chemical structure of AF, which requires long multistep synthetic strategies, results in high synthesis cost and limited access to synthetic analogues [11]. But secondly, and most importantly, the 3D structure of AOX was unknown until recently [21], therefore not much was known about the protein–ligand interactions, which hindered a rational strategy towards the design of potent inhibitors. Upon elucidation of TAO’s crystal structure, structure-aided design of TAO inhibitors became possible and, since then, several structure–activity relationship (SAR) analyses have been performed [17, 22].

The aim of our investigation was twofold: (1) to elucidate the detailed mode of interaction of TAO and its inhibitors in order to identify key interactions that are required to lead to high potency of inhibitors, and (2) to identify molecular characteristics in inhibitors that contribute to the inhibitory activity towards TAO. These aspects are important for the discovery of novel, potent TAO inhibitors. In order to achieve these goals, a reliable set of inhibitors with consistently measured activity is required. Furthermore, an external set of compounds are needed to assess the validity of any models. Saimoto et al*.* recently synthesized several AF derivatives, for which they experimentally obtained the 50% inhibitory concentration (IC_50_) [17]. This dataset provides an excellent resource for the development of models and docking studies and was used here as the training set. Moreover, we measured the IC_50_ values of a different set of compounds and used these as the test set to assess the validity of the developed models. To identify the binding interactions occurring between the inhibitors and TAO, and to further understand their inhibitory mechanism, we used the only AOX crystal structure available to date, which is the trypanosomal alternative oxidase of *Trypanosoma brucei* [21]. Molecular docking studies, combined with Quantitative Structure–Activity Relationship (QSAR) analyses, were used to offer insight into understanding the details of protein-inhibitor interactions and the factors affecting bioactivity, providing information for the design of new drug candidates and prediction of inhibitory activity of unknown compounds. Herein, we present a computational study, in which we predict the most favorable binding mode and interaction mechanism between experimentally identified TAO inhibitors and the trypanosome alternative oxidase using molecular docking and QSAR. QSAR analysis correlates the experimentally obtained IC_50_ values with computed molecular properties such as molecular descriptors and fingerprints, in order to investigate and highlight the structural features responsible for the AF derivatives’ inhibitory activity. Overall, this analysis provides significant information about the structural features responsible for the interaction between the inhibitors and TAO and their mechanism of inhibition.

## Materials and methods

### Materials

All chemicals were purchased from Sigma-Aldrich unless otherwise indicated and were of the highest purity available. Ascofuranone, ascochlorin and ferulenol were kindly provided by Professor Kita and Dr. K. D. Inaoka (University of Nagasaki), whereas colletochlorin B and colletochlorin D were synthesised in-house [23].

### High resolution respirometry

Respiratory activity of recombinant E. c*oli* membranes expressing TAO were monitored with high-resolution respirometry to experimentally determine the IC_50_ values of 8 AOX inhibitors (Fig. 1). Respiration was monitored using an Oroboros® Oxygraph-2K (Oroboros Instruments, Innsbruck, Austria). Data acquisition and analysis were performed using DatLab® software, version 6.1 (Oroboros Instruments) and GraphPad Prism 7.

Methods for *E. coli* membrane preparations have been presented before [23]. For the trypanosome alternative oxidase inhibition assay, the O2k chambers were calibrated for 2 ml of 65 mM MOPS buffer adjusted to pH 7.4 and equilibrated with air at 37 °C at the beginning of each experiment. TAO membrane bounds (~ 600 µg total protein) were added to closed chambers containing respiration buffer with 1 mM KCN and supplemented with 1.25 mM NADH as substrate to assess basal respiration. Effects of increasing concentrations of inhibitor were determined using a single sample. Average values from 3 isolations ± standard deviation where applicable.

### Datasets for QSAR and docking

**Fig. 2:**
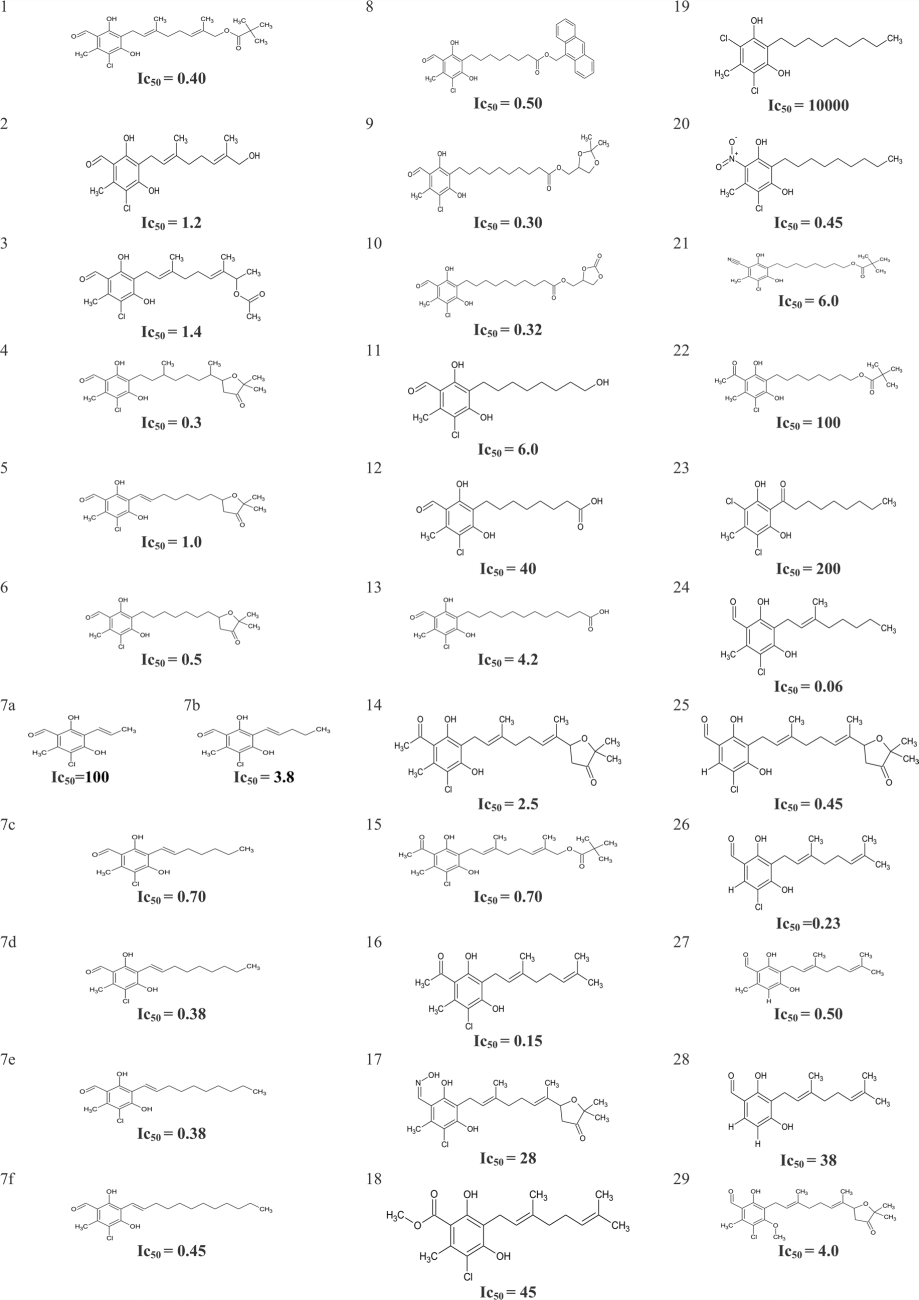
Structures and 50% inhibitory concentrations (nM) of a series of AF derivatives obtained by Saimoto et al. [17]

A series of 34 Ascofuranone (AF) derivatives synthesized by Saimoto et al. [17] with inhibitory activities against the alternative oxidase of *Trypanosoma brucei* were taken to perform the in silico study. The molecular structures of these compounds along with their respective 50% inhibitory concentrations (IC_50_) against TAO are shown Fig. 2. For the development of QSAR models, IC_50_ values were converted to the logarithmic scale (pIC_50_ values, calculated as -log IC_50_) to ensure normal distribution in statistical analyses, and these were used as the dependent variable in the QSAR analyses.

In addition to the 34 AF derivatives, IC_50_ values of a collection of 8 AOX inhibitors (Fig. 1) were measured using high resolution respirometry, and these were used as our external test compounds.

The chemical structures of all compounds used in this study were drawn with ACD/ChemSketch 2016.2 software and their SMILES (Simplified Molecular Input Line Entry Specification) notations were generated for future uses in molecular modelling and QSAR software.

### Molecular docking

#### Structure preparation

The crystal structure of the cyanide-insensitive alternative oxidase from *Trypanosoma brucei* (TAO) was obtained from RCSB Protein Data Bank (PDB entry: 3W54). The protein structure was loaded into MOE software (Molecular Operating Environment, version 2016.08, Chemical Computing Group Inc., Montreal, Canada) to carry out some preparatory steps. Hence, the hydrogen bond network was optimised and protonation of amino acid residues was corrected using the Protonate 3D algorithm, while AMBER99 forcefield was used to assign correct atomic charges.

The SMILES codes of the compounds were used to input the structures into MOE, where the 3D structures were developed. The molecules were then washed and partial atomic charges were assigned. Following this, the molecules were subjected to an initial energy minimisation using the AMBER10:EHT forcefield, followed by a second minimisation using the MOPAC semi-empirical energy function (PM3 Hamiltonian).

#### Docking of the inhibitors

Molecular docking studies were performed using the MOE software package. All crystallised water molecules and coordinated molecules present in the TAO structure were preserved. The binding site of TAO was defined using colletochlorin B, the native ligand crystallised in the structure. Hence, the inhibitors were docked into the binding site of this crystallographic ligand. In the MOE dock panel, the placement method used was Triangle Matcher, and the poses generated by this methodology were re-scored using London dG scoring (Eq. 1). Subsequently, poses resulting from the placement stage were further refined using the Induced Fit method, which allows protein flexibility upon ligand binding, improving the prediction accuracy for the interaction. Poses were then rescored using the GBVI/WSA dG scoring function (Eq. 2), and the top five best scoring poses were retained. The final output was analysed, and docked poses that were not correctly orientated (for catalytic site) within the binding site were discarded and not included in the analysis.

London dG scoring: The London dG score estimates binding free energy using:

1 $$\Delta G = c + E_{flex} + \mathop \sum \limits_{h - bonds} C_{HB} f_{HB} + \mathop \sum \limits_{m - lig} C_{M} f_{M} + \mathop \sum \limits_{atoms i} \Delta D_{i} ,$$

where c is the average gain/loss of rotational and translational entropy, $E_{flex}$ is the energy loss due to ligand flexibility, $f_{HB}$ measures H-bond geometric imperfections and takes a value [0,1], $C_{HB}$ is the ideal H-bond energy, $f_{M}$ measures metal ligations geometric imperfections [0,1], $C_{M}$ is the energy of an ideal metal ligation and $D_{i}$ is the desolvation energy of atom i.

GBVI/WSA dG scoring: The GBVI/WSA dG score estimates binding free energy by:

2 $$\Delta G \approx c + \alpha \frac{2}{3}\left( {\Delta E_{Coul} + \Delta E_{sol} } \right) + \Delta E_{vdW} + \beta \Delta SA_{weighted} ,$$

where c is the average gain/loss of rotational and translational entropy, α and β are equation constants determined during training, $E_{Coul}$ is the coulombic electrostatic energy, $E_{sol}$ is the solvation electrostatic term, $E_{vdW}$ is the van der Waals energy and $SA_{weighted}$ is the weighted surface area.

### QSAR model

#### Calculation of molecular descriptors

Molecular descriptors for the 34 AF derivatives and for the external test compounds were calculated using Advanced Chemistry Development (ACD/Labs) Percepta version 2016.1 and MOE software (QuaSAR descriptor panel). Initially, in order to reduce the number of non-significant variables, descriptors with missing values (> 10%) or those with > 97% of constant values were eliminated from the data set. Additionally, the best pose docking scores and the average docking scores of the retained top three and top five poses for each compound were used as additional molecular descriptors for the analysis. Furthermore, an indicator variable was devised which indicated the presence (value 1) or absence (value 0) of a hydrogen bond acceptor group in the meta position of the aromatic ring with respect to the longest hydrocarbon chain on the ring. This indicator variable was used as an additional molecular descriptor in QSAR analysis. As a result, a total of 331 predictors (molecular descriptors) were used as independent variables in statistical analyses.

#### Model development and validation

For the development of QSAR models, only the 34 AF derivatives were used as the training set and models were developed using stepwise regression analysis as below.

##### Bagging and stepwise regression analysis for regression model

To prune the number of initial molecular descriptors and select the predominant ones affecting the inhibitory activity of the AF derivatives against the trypanosomal alternative oxidase, a bagging algorithm combined with stepwise regression analysis was performed using IBM SPSS Statistics software, version 24.

In order to perform bagging for feature selection and to identify the most important descriptors correlating with the pIC_50_ values, random allocation of compounds into various subsets of training set was carried out, where each training subset comprised ~ 80% of total number of compounds. To this end, MatLab® was used to carry out a random permutation to obtain 85 subsets of different combinations of compounds (85 training subsets). In these permutations, 7 compounds of the total set (~ 20%) were eliminated randomly each time in order to be used as the “internal validation subset”, whereas the remaining 27 compounds (~ 80%) were kept to be used as the “training subset”. Stepwise regression analysis was carried out for each training subset (85 times) and the three most important molecular descriptors were selected in each analysis. Stepwise regression analysis is a linear feature selection method in which a model is built by successively adding or removing predictor variables (molecular descriptors), generating a multiple linear regression equation that includes the variables that best explain the distribution of the dependent variable. The stepping criteria was set to default (‘alpha to enter’ = 0.05 and ‘alpha to remove’ = 0.10).A collection of these selected descriptors (48 descriptors in total) were used to perform the final stepwise regression analysis using all 34 compounds, from which top four best molecular descriptors were identified. The optimal QSAR model was then obtained by multiple regression analysis in IBM SPSS Statistics software using the top four best descriptors of the final stepwise regression analysis. For a statistically reliable model, we maintained a ratio of at least 5:1 between the number of compounds and number of descriptors used.

##### Pre-processing feature selection for regression model

An additional “independent” feature selection was performed using Waikato Environment for Knowledge Analysis (Weka) machine learning software. The attribute evaluator was WrapperSubsetEval with Random Forest, and the search method was GeneticSearch. All the default settings for GeneticSearch and Random Forest were used, but the maximum depth of the trees in random forest was limited to 4 (MaxDepth = 4) to correspond to the maximum number of molecular descriptors we allowed in all other analyses. Following this feature selection, stepwise regression analysis was performed using the selected set of molecular descriptors.

##### Model validation

The final QSAR models were validated internally and externally. The internal validation procedure used was leave-one-out (LOO), as well as leave-some-out (LSO) cross validation. For LSO method, compounds were divided into 5 groups, each group was removed once, and the multiple regression was performed using the remaining compounds. The predicted values for the internal validation set were collected, and Mean Absolute Error (MAE) as well as the cross-validated R^2^ (Q^2^) were used to assess the accuracy of prediction of pIC_50_ (Eqs. 3 and 4).

Cross-validated R^2^ (Q^2^):

3 $$Q^{2} = 1 - \frac{{\sum \left( {Y_{obs } - Y_{pred} } \right)^{2} }}{{\sum \left( {Y_{obs} - \overline{Y}_{pred} } \right)^{2} }}$$

where $Y_{obs}$ and $Y_{pred}$ correspond to the observed and LOO/LSO predicted pIC_50_ value, respectively.$\overline{Y}_{pred}$ refers to the average of the predicted pIC_50_ values based on the LOO/LSO technique.

Mean Absolute Error (MAE):

4 $$MAE = \frac{{\sum \left( {\left| {Y_{obs} - Y_{pred} } \right|} \right)}}{N}$$

where $Y_{obs}$ refers to the experimentally obtained pIC_50_ value, $Y_{pred}$ is the predicted pIC_50_ value for the internal validation set by the QSAR model, and N is the number of compounds.

The external validation was done by using the external test compounds, which are represented by the 8 specific AOX inhibitors that were not used to develop the model (Fig. 1). pIC_50_ values for each compound were predicted using the QSAR model to examine its consistency and capability of predicting. Then the mean absolute error (MAE) was calculated for the external test set. Furthermore, the square correlation coefficient (R^2^) between the observed and the predicted activity values, another parameter proposed by Golbraikh and Tropsha for determining the external predictability of a QSAR model, was calculated. According to these authors, a QSAR model is considered predictive if R^2^ > 0.6 [24].

##### Y-Randomisation

For Y-Randomisation, 100 permutations of pIC_50_ values were performed and “fake” QSARs were developed for each of these 100 randomized vectors using exact same methods described above. This included: (1) bagging with stepwise regression for feature selection, followed by stepwise regression analysis, and (2) feature selection using WrapperSubsetEval coupled with Random Forest with GeneticSearch method, followed by stepwise regression analysis.

## Results

### Molecular docking analysis

#### Validation of docking using binding mode of colletochlorin B

**Fig. 3:**
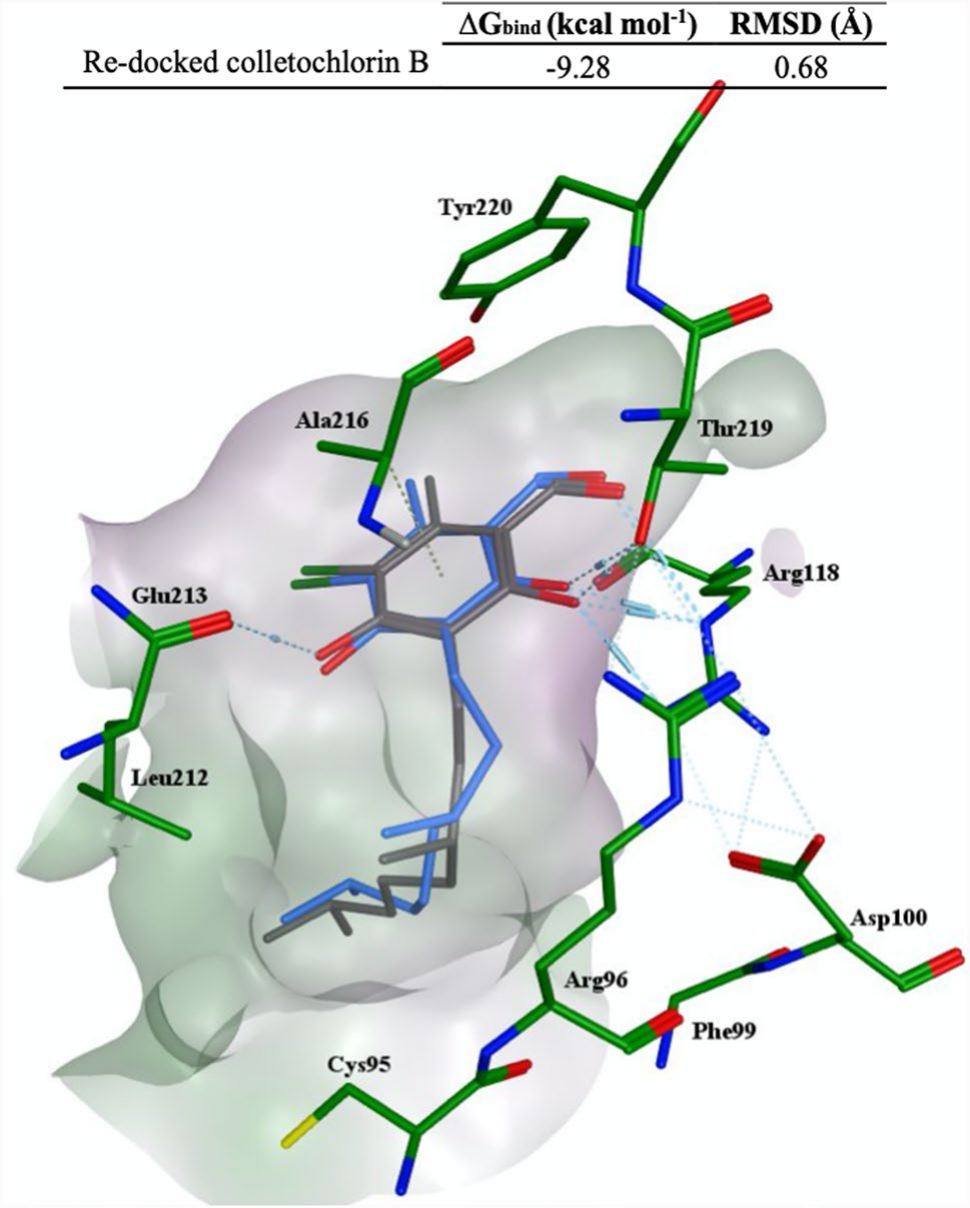
Superimposition of the crystallographic colletochlorin B (carbon atoms in grey) and the best ranked pose of the re-docked colletochlorin B (carbon atoms in blue). Hydrogen bonds are represented by light blue dashed lines. Oxygen, nitrogen and sulfur atoms are depicted in red, blue and yellow, respectively

To assess the reliability of the docking procedure used, the co-crystallized colletochlorin B (CB) was redocked into the binding site of TAO. As a result, the root mean square deviation (RMSD) between the crystallographic conformation and the re-docked conformation of colletochlorin B was 0.68 Å (Fig. 3), suggesting an acceptable accuracy for the docking procedure to predict the binding mode of the TAO inhibitors. This is a satisfactory value that may suggest that the docking method could be valid for the inhibitors studied in this manuscript.

**Table:** 

	Calculated ∆G_bind_ (kcal mol^−1^)	RMSD (Å)
Re-docked colletochlorin B	− 9.28	0.68

#### Interaction fingerprint analysis by PLIF

**Fig. 4:**
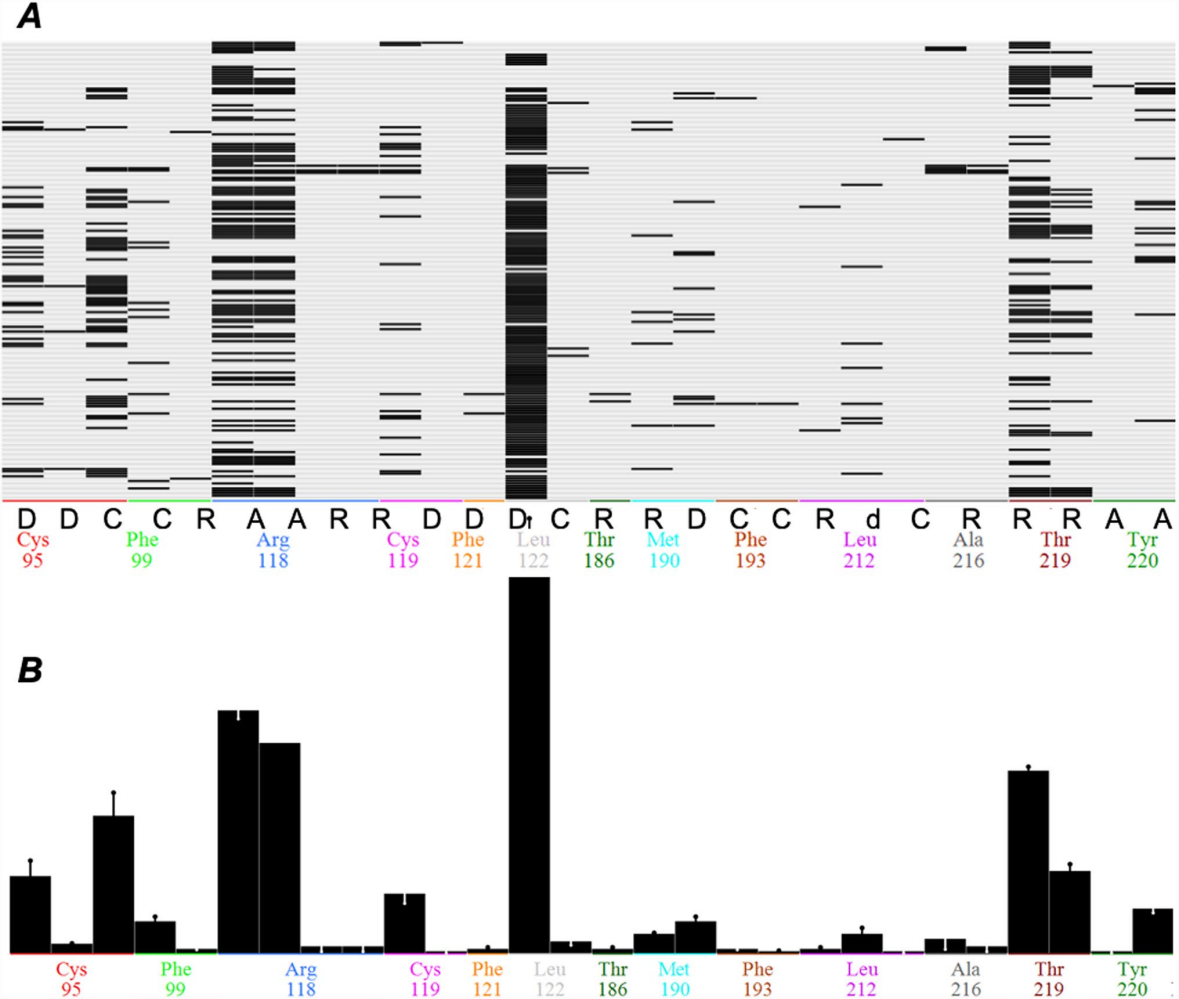
**a** Interaction fingerprint matrix. The columns represent interactions, which are labelled with the residues (coded with an arbitrary color) and corresponding type of interaction. Encoded fingerprints are present as black rectangles. The rows represent the top five ranked poses for each inhibitor. **b** The population histogram shows the frequency of occurrence amongst the inhibitors for each of the selected fingerprint bits, i.e. the number of inhibitors with which each residue interacts. The X-axis is constructed in the same way as for the interaction matrix, while the Y-axis shows a black bar which shows the relative counts for the bits

In this study, the 34 AF derivatives reported by Saimoto et al. [17], along with 8 specific AOX inhibitors with IC_50_ values measured here (Fig. 1) were docked into the binding site of TAO. Based on the docking results, an interaction analysis was performed by protein–ligand interaction fingerprinting (PLIF) to provide detailed information on protein–ligand interactions. The PLIF tool within MOE summarizes the interactions of the compounds with the AOX ligand binding residues using a fingerprint scheme, in which interactions are classified according to the residue of origin (Fig. 4). There are several types of interactions in which a residue may participate: sidechain hydrogen bonds (donor or acceptor), backbone hydrogen bonds (donor or acceptor), solvent hydrogen bonds (donor or acceptor), ionic interactions, surface interactions, metal binding interactions and arene interactions. The fingerprints present within the inhibitor-AOX complexes include surface contact interactions (C), backbone hydrogen bond donors (d), sidechain hydrogen bond acceptors (A) and donors (D) and arene interactions (R) (Fig. 4A). The bit selector tool within MOE can provide detailed information about the nature and statistics of each fingerprint. The percentage abundance of each fingerprint is shown in Table 1. It is calculated in a way that if the fingerprint bit occurs in every entry, the % abundance will be 100. The residues that interact most often with the ligands are: leucine 122 (C) (80.95%), arginine 118 (A) (46.66% and 40%), threonine 219 (A) (34.76% and 15.71%) and cysteine 95 (C, D) (26.19% and 14.76%).

**Table 1:** Percentage abundance of the fingerprint bit throughout the database

Residue	Type of interaction	% Abundance	Strength of Interaction
Leu 122	Surface contact	80.95	
Arg 118	Sidechain hydrogen bond acceptor	46.66	Strong (1.5 kcal/mol)
Arg 118	Sidechain hydrogen bond acceptor	40.00	Weak (0.5 kcal/mol)
Thr 219	Sidechain hydrogen bond acceptor	34.76	Weak (0.5 kcal/mol)
Cys 95	Surface contact	26.19	
Thr 219	Sidechain hydrogen bond acceptor	15.71	Strong (1.5 kcal/mol)
Cys 95	Sidechain hydrogen bond donor	14.76	Weak (0.5 kcal/mol)
Cys 119	Sidechain hydrogen bond donor	11.90	Weak (0.5 kcal/mol)
Tyr 220	Arene interaction	9.04	

To better understand the interaction mechanism, we investigated the significance of the fingerprints for a compound to be an active inhibitor. For this purpose, all compounds were classified as active or inactive based on their IC_50_ values. Because 76% of the IC_50_s of the AF derivatives reported by Saimoto et al. ranged between 0.06 to 6 nM [17], we decided to set up the threshold at 10 nM, with compounds having IC_50_ ≥ 10 nM considered as inactive. In Fig. 4B, the frequency bars are annotated with a qualitative indication of the overabundance of active with the residue interaction vs. overall number of active compounds. The extent to which the fraction of actives containing the bit is higher than the fraction of actives overall is indicated as a black line above the bar. If, on the contrary, the fraction of actives containing the bit is lower than the fraction of actives overall, it will be indicated as an inverse (white) line below the top of the bar. Generally, the combination of height and overabundance is often an indication that the corresponding interaction is important for activity.

#### Binding mode of the inhibitors

**Fig. 5:**
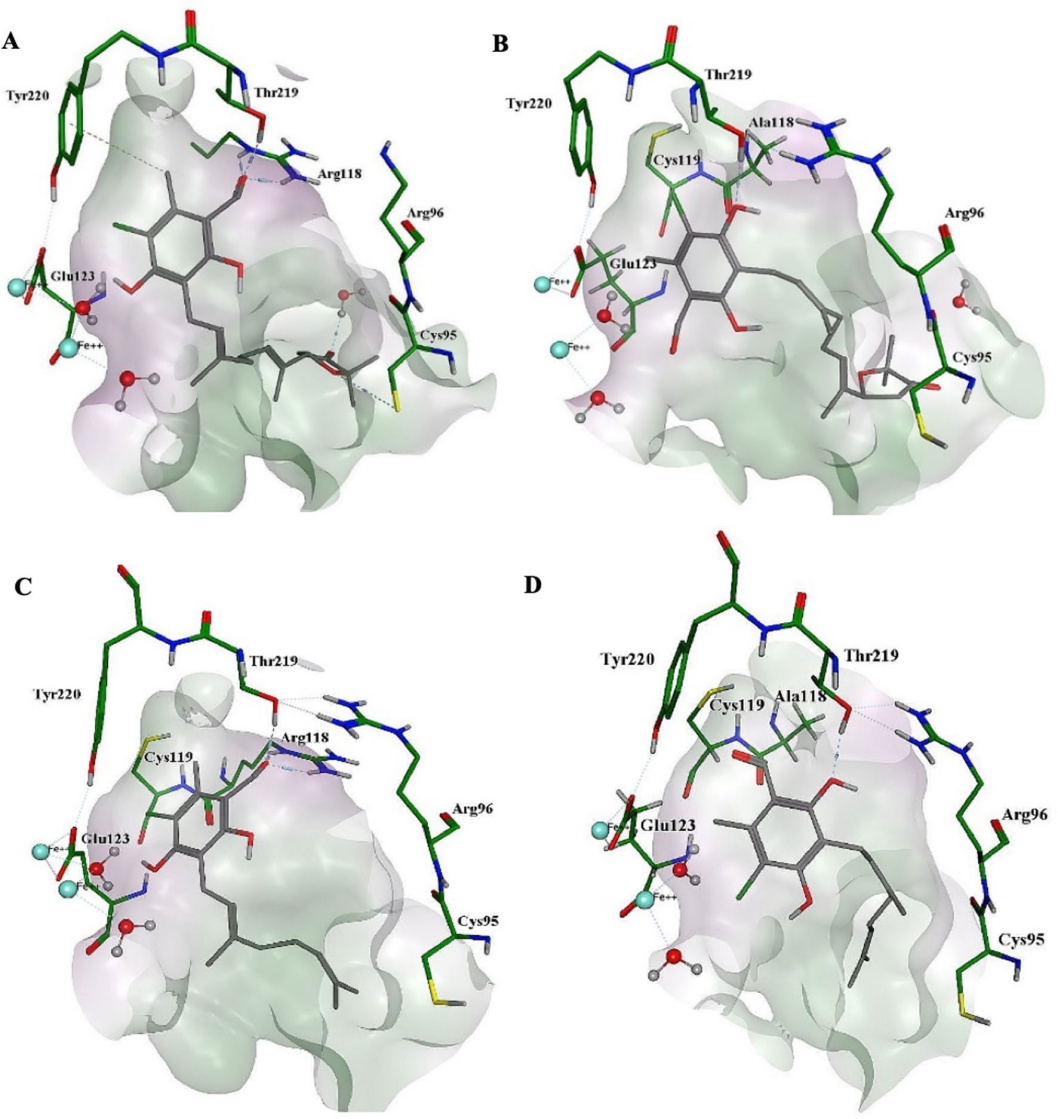
Predicted docked poses for inhibitors within the binding site of TAO (PDB: 3W54). **a** Ascofuranone and wild type TAO. **b** Ascofuranone and R118A mutated TAO. **c** Colletochlorin B and wild type TAO. **d** Colletochlorin B and R118A mutated TAO. The molecular surface is indicated in green for hydrophobic regions and pink for hydrophilic regions. Hydrogen bonds are represented by blue dashed lines. Arene-H interaction is represented by green dotted line. Diiron centre is represented by turquoise spheres. Oxygen, nitrogen and sulfur atoms are depicted in red, blue and yellow, respectively

Docking poses of the inhibitors within TAO were compared with those with point mutated TAO, where Arg 118 was replaced with alanine. Figure 5 shows the top poses of ascofuranone and colletochlorin B docked into the binding site of the wild type and mutated TAO.

The active site of AOX is composed of a diiron centre, four glutamates (Glu 123, Glu 162, Glu 213 and Glu 266) and two histidine residues (His 165 and His 269), all of which are fully conserved [2]. Tyrosine residues also have a key role in the catalytic activity of AOX. For instance, Tyr 220, which is also identified in our PLIF analysis to interact with several inhibitors, is totally conserved across all AOXs, and is buried within 4 Ǻ of the diiron centre (Fig. 5) [1, 2, 21, 25]. In addition to the catalytic site, the AOX contains a binding site for its natural substrate ubiquinol. For docking analysis, this site was defined using the co-crystallised colletochlorin B depicted in the crystal structure. From the results obtained, it is apparent that the inhibitors bind to the enzyme in a manner that the aromatic head remains close to the diiron center, as well as to the residues Arg 118, Thr 219 and Arg 96. This seems to be in agreement with the binding mode of colletochlorin B to TAO previously reported by Shiba et al. [21]. Residues Arg 118 and Thr 219 are of particular importance, as they form hydrogen bonds with the functional groups on the aromatic ring of the inhibitors, establishing a strong interaction between the compounds and the protein. These hydrogen bonds are key for the potent inhibitory activities of the inhibitors [17], which is also shown from the results of PLIF analysis (Fig. 4).

Figure 5 depicts ascofuranone and colletochlorin B within the hydrophobic pocket of TAO. These two are phenolic acids, with 5-chloro orsellinaldehyde structures (see Fig. 1). In the wild type TAO (Figs. 5a, c), the key interactions of these ligands with the protein occur through hydrogen bonding between the formyl group of the orsellinaldehyde and Thr 219 and Arg 118 residues. The 4-OH of the orsellinaldehyde group, on the other hand, is seen to be interacting with the diiron centre. However, when the Arg 118 is mutated to alanine (Fig. 5b, d), the hydrogen bond formations are lost for both inhibitors, displacing the compounds away from the diiron centre.

Similar docking experiments were performed with two other TAO mutants, L122A and T219A. As it can be seen from the docking scores obtained (Table 2), the importance of these residues was confirmed by comparison of the docking scores with the wild type TAO. As would be expected, mutations on these particular residues cause the docking scores to increase, indicating lower affinity and less favorable interactions. Furthermore, results show that L122A and R118A mutations have a more profound effect on binding affinity than T219A mutations [26].

**Table 2:** Docking scores of ubiquinol (natural substrate of TAO), some of the most potent and novel TAO specific inhibitors (AF, CB, compounds 16 and 24—see Fig. 2 for the chemical structures) and the classic inhibitors SHAM and propyl gallate against wild type TAO and specific point mutants

Compound	Calculated ∆G_bind_ (kcal mol^−1^)				IC_50_ (nM)
	Wild type	L122A	R118A	T219A	
Ubiquinol	− 9.77	− 8.36	− 8.87	− 9.18	
Ascofuranone	− 9.65	− 8.92	− 8.89	− 9.50	0.13
Colletochlorin B	− 8.76	− 8.06	− 7.94	− 8.70	0.20
16	− 9.21	− 8.47	− 8.58	− 8.78	0.15
24	− 8.52	− 7.92	− 7.72	− 8.42	0.06
SHAM	− 5.26	− 5.48	− 5.15	− 5.35	4000
Propyl gallate	− 6.67	− 6.43	− 6.43	− 6.46	200

Calculated binding free energy is depicted in kcal mol^−1^ and IC_50_ in nM. IC_50_ values of wild type TAO were obtained by Saimoto et al. [17].

The extra hydrogen bond acceptors on the furanone ring of ascofuranone (Fig. 1) do not appear to interact with any side chains in the majority of the determined poses, the most likely H-bond formation being with the Cys 95 backbone as shown in Fig. 5a. Given the lack of improvement of inhibition by ascofuranone over colletochlorin B, it is unlikely that this extra hydrogen bond plays a significant role in the efficacy of inhibition under the experimental conditions defined in this study.

As can be seen in Table 2, docking scores (kcal mol^−1^) rank correlate well with the potency of the inhibitors, which shows the docking procedure provides realistic results. For instance, ubiquinol, the natural substrate of TAO, gives the lowest docking score in the wild type (− 9.77 kcal mol^−1^), followed closely by ascofuranone, the most potent inhibitor of TAO (− 9.65 kcal mol^−1^). SHAM or propyl gallate, on the other hand, are the least potent inhibitors, which is reflected in their high docking scores (− 5.26 and − 6.67 kcal mol^−1^, respectively).

The two mutations that seemed to affect the final docking scores the most were L122A and R118A, which is in agreement with the PLIF analysis shown in Table 1 that indicated that these were the residues that compounds interacted the most with (Table 1). Interestingly, mutations of these key residues seemed to have a greater impact on the interactions of potent inhibitors (such as AF, CB, compound 16 and 24) than on the the classic inhibitors such as SHAM or propyl gallate. In summary, all the mutations that were performed (L122A, R118A, T219A) caused all scores to increase (except for L122A and T219A in the case of the weakest inhibitor, SHAM), which proves their key role in the interactions between inhibitors and the enzyme [26].

### QSAR models

For the 34 AF derivatives, a QSAR model was developed using the molecular descriptors selected by stepwise regression analysis as explained in the methods section. The obtained linear model consists of four molecular descriptors which have been picked out of the initial pool of 331, as shown below.

$$pIC50 = - 0.124 + 2.252\,metaHacceptor - 2.527\,Neutral\;form \, (pH \, 7.4){-} \, 18.014\,PEOE\_VSA\_FPPOS + \, 17.527\,petitjean$$

$${\text{n}} = {34};\;{\text{R}}^{{2}} = 0.{72}0;\;{\text{s}} = 0.{632};\;{\text{F}} = {18}.{6}0{3};\;{\text{p}} = 0.000$$

Here, n is the number of compounds used in the development of the QSAR model; R^2^ is the squared correlation coefficient; s is the standard error of the estimate, F is the Fischer ratio and p is the statistical confidence level. All regression coefficients are significant at p < 0.05.

The first molecular descriptor selected by the analysis is *metaH-acceptor,* which is an indicator variable showing the presence or absence of hydrogen bond acceptor group(s) in meta position with respect to the long alkyl side substituent of the benzene ring (corresponding to positions 1 or 5 of the orsellinaldehyde group as shown for ascofuranone in Fig. 1). This descriptor takes binary values (0, 1) to indicate the absence or the presence of the hydrogen bond acceptor property, respectively. It must be noted that this H-bonding group is in addition to the phenolic hydroxyl groups in ortho position(s) (corresponding to positions 2 and 4 in ascofuranone in Fig. 1). The positive coefficient of *metaH-acceptor* in the equation indicates that the presence of such groups in meta position provides greater ability for the compounds to interact more effectively with the protein. This agrees well with the docking results discussed earlier regarding the role of hydrogen bonding groups in this position, e.g. formyl group of ascofuranone, for interaction of inhibitors with Arg 118 and Thr 219. This is consistent with the fact that hydrogen bonds are key for strong interactions in the protein–ligand complexes. Accordingly, compounds that lack hydrogen bond acceptor groups in meta positions (compounds 19 and 23) show the lowest pIC_50_ values (Fig. 2).

Furthermore, the second most important descriptor in the equation is *Neutral form*. This is the fraction of molecules that is unionised in acid/base protonations at pH 7.4, calculated by the classic ACD Percepta method. Given that the compounds in the training set do not possess any basic groups and only acid dissociation is possible for some of them, the negative coefficient of *Neutral form* indicates that the more acidic compounds (with lower unionized fractions) are better TAO inhibitors with higher pIC_50_ values. The acidity of these compounds, with two exceptions, is from the ortho-phenolic hydroxyl groups, corresponding to positions 2 and 4 in ascofuranone in Fig. 1. The exceptions are compounds 12 and 13 which have a more acidic carboxylic acid group at one end of the linker group. Given that according to the third molecular descriptor in the equation (*PEOE_VSA_FPPOS*), presence of carboxylic acid groups on the linker group has a negative impact on pIC_50_ (as described in the next paragraph), it can be concluded that it is only the acidity of the ortho-phenolic groups that leads to increased potency. This finding is also consistent with the docking results that show a hydrogen bonding between Glu123 and the phenolic hydroxyl group (see Figs. 3 and 5).

The third most important molecular descriptor in the equation is *PEOE_VSA_FPPOS*. It is a charge-dependent molecular descriptor where atomic charge is calculated through the Partial Equalization of Orbital Electronegativities PEOE method [27]. This molecular descriptor represents the fractional positive polar van der Waals surface area. Based on the negative coefficient of this parameter in the equation, compounds with lower fraction of positively charged atoms, *i.e.* larger molecules with fewer (electronegative) heteroatoms, will present higher pIC_50_ values and a greater efficacy in inhibiting TAO. Within the dataset, all compounds contain an aromatic group with phenolic hydroxyl(s) and a linker (alkyl side chain) group, with some compounds containing an additional carboxylic acid group attached to the other end of this linker. Presence of such carboxylic acid groups (*i.e.* high values of *PEOE_VSA_FPPOS)* reduces the inhibition potency (e.g. compounds 12 and 13, see Supplementary data, Fig. 2).

Finally, the last molecular descriptor selected in the model is *petitjean,* which is a “shape coefficient”. This molecular descriptor is defined as the ratio (D-R)/R, where R is the generalised radius and D is the generalised diameter calculated using graph theoretical methods in MOE software [28]. Larger *petitjean* values in the AF derivatives correspond to more linear (longer linker chain) molecules, which according to the positive sign of this descriptor in the equation, will result in higher pIC_50_ values. In contrast, compounds with shorter linker chains (e.g. compounds 7a, 7b and 7c, see Supplementary data, Fig. 2) have the lowest *petitjean* values.

**Fig. 6:**
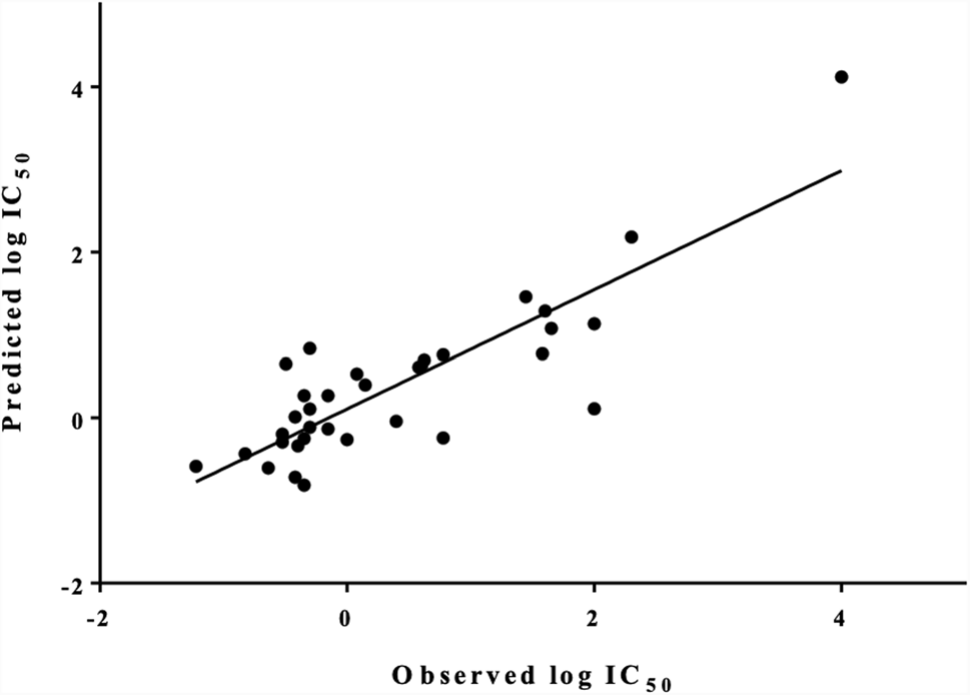
Predicted pIC_50_ values with the QSAR model vs. experimentally obtained values (training set). Pearson correlation (R): 0.719, p < 0.0001 (95% confidence interval). MAE was 0.417

The linear plot of the predicted pIC_50_ values based on the QSAR model developed in this study versus the experimental values is shown in Fig. 6.

#### Investigating the risk of chance correlation

Due to the fact that this data is high-dimensional, i.e. only 34 compounds compared with a total of 331 molecular descriptors, there is a need to investigate the risk of chance correlations. The high number of molecular descriptors could still be a problem despite reducing this initial high number of molecular descriptors to 48 by using the bagging method as described in the the methods section [29, 30]. Two steps were taken in order to assess the risk of chance correlations and identify whether the selected molecular descriptors are in fact meaningful in the prediction of pIC_50_ values, and not a mere chance correlation. These were y-Randomisation test and performing an independent feature selection to compare the results with this current model.

Y-Randomisation test can indicate the chance of obtaining a good correlation when there is in fact no cause-effect relationship due to Y values being randomly ordered. If the statistical results are poorer in randomised models than those of the actual model, then it can be concluded with reasonable confidence that the actual model is not obtained by any chance. For y-Randomisation, we performed 100 permutations of pIC_50_ values, and then developed QSARs for these randomised vectors using the exact same procedure as with the “real” QSAR, *i.e.* bagging for feature selection followed by stepwise regression analysis. The results showed that 89% and 95% of these “fake” QSAR models had Q^2^ values < 0.4 and < 0.5, respectively. There was one fake model that had a Q^2^ > 0.6 (to be exact, R^2^ = 0.685 and Q^2^ = 0.629 for this fake model compared with the real model with R^2^ = 0.720 and Q^2^ = 0.637). To account for the impact of correlations between the randomised vectors and the original pIC_50_ vector, y-Randomisation plots between this correlation coefficient, r, and Q^2^ or R^2^ of all the 100 randomised fake models and the real QSAR were drawn. The linear regression lines had a R^2^_Y_ intercept of 0.36 and a Q^2^_Y_ intercept of 0.11 (See Supplementary data, Figure S1), while the recommendation for a “chance free” QSAR model is that these values should not exceed 0.3 and 0.05 [31].

In the second experiment, an “independent” pre-processing feature selection strategy was performed in Weka, as described in the methods section. This method selected 67 molecular descriptors for pIC_50_ and between 8–170 (with an average of 83) molecular descriptors for each of the 100 permutations of pIC_50_. Next, stepwise regression analysis was used to develop QSAR equations for pIC_50_ and the randomised pIC_50_ vectors, while the maximum number of molecular descriptors in the equations were limited to 4 (as before). In this case, 93% and 98% of these “fake” QSAR models had Q^2^ values < 0.4 and < 0.5, respectively, and none had a Q^2^ > 0.6. Of the randomised pIC_50_s, 20 did not show any correlation at all. The y-Randomisation plot had a R^2^_Y_ intercept of 0.23 and Q^2^_Y_ intercept of 0.02, which follows the recommendation for chance free QSAR (See Supplementary data, Fig. S2). As shown below, this new QSAR for pIC_50_ is similar to the original in terms of the first two molecular descriptors and has slightly lower statistical properties.

$$pIC50 = 11.520 + 1.902\,metaHacceptor - 2.803\,Neutral\;form \, (pH \, 7.4) + \, 13.366\,GCUT\_PEOE\_1 + \, 0.106\,opr\_brigid$$

$${\text{n}} = {34};\;{\text{R}}^{{2}} = 0.{7}0{5};\;{\text{s}} = 0.{649};\;{\text{F}} = {17}.{333};\;{\text{p}} = 0.000;\;{\text{Q}}^{{2}} = 0.{6}0{1}$$

The fact that the first two molecular descriptors here are the same as the previous equation adds to the evidence that these two molecular properties are involved in the AOX inhibitory potency. The third molecular descriptor, *GCUT_PEOE_1*, is a graph theoretical molecular descriptor, as is *petitjean* in the previous QSAR. It is calculated from the eigenvalues of a modified graph distance adjacency matrix, where the diagonal takes the value of the PEOE partial charges. It relates to the distribution of partial atomic charges in the molecular structure, where in this series of molecules, larger *GCUT_PEOE_1* values are observed with the presence of bulky hydrocarbon linker group (e.g. compounds 1, 15, 16 and 27), while the lowest values correspond to shorter unbranched linker groups (see compounds 7a, 8 and 12). The last molecular descriptor here, *opr_brigid*, is the number of rigid bonds including ring bonds and double bonds [32]; compounds with an additional ring attached to the linker group (e.g. compounds 5 and 8) have higher *opr_brigid* values with a positive impact on their pIC_50_ values.

The description of the last 2 molecular descriptors of these two QSAR equations is summarised below to facilitate the comparison of the two equations. Table 3 shows that also based on the last two molecular descriptors, both QSAR models come to the same conclusion regarding the effect of size and the length of the linker group. However, the latter QSAR misses a molecular description of the effect of polarity.

**Table 3:** Molecular properties for higher pIC_50_ values

QSAR 1	QSAR 2
*PEOE_VSA_FPPOS*: larger compounds with fewer heteroatoms	*GCUT_PEOE_1*: bulky hydrocarbon linker groups
*Petitjean*: longer linker groups	*opr_brigid*: more rings

#### Internal and external validation of the QSAR model

**Fig. 7:**
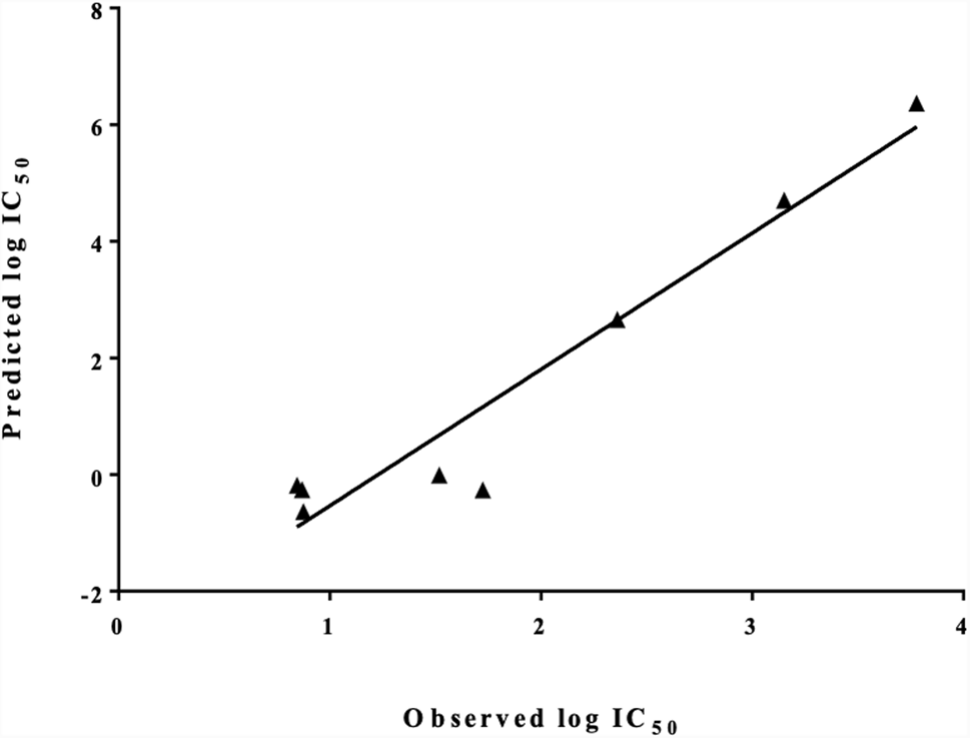
Predicted pIC_50_ values of external test compounds with QSAR model vs. experimentally obtained ones. Pearson correlation (R): 0.903, p value = 0.0001 (95% confidence interval)

The QSAR model was first internally validated to evaluate its accuracy. As described in the materials and methods section, four sets of 6 or 7 compounds were removed from the dataset; QSAR model was then rebuilt based on the remaining compounds, and the activity of the deleted compounds was predicted based on the resulting QSAR equation. In this exercise, every compound was left out once. Mean absolute error (MAE) of predicted pIC_50_ values for the validation set was 0.485 and Q^2^ value was 0.637. The leave-one-out (LOO) cross-validation was also performed where all the compounds are removed once, one at a time, and the QSAR model is rebuilt based on the remaining molecules. The activity of the deleted compounds is then calculated based on the resulting QSAR equation. The LOO Q^2^ was 0.623, while MAE of predicted pIC_50_ values with the LOO cross-validation method was 0.487. According to Tropsha, a QSAR model is considered acceptable if the value of Q^2^ exceeds the predetermined value of 0.5 [33] (see Supplementary data, Table S1 for individual absolute error values for each compound). Based on the mean absolute error obtained through internal validation, we can judge the goodness-of-fit of the model. However, this validation approach lacks predictability when the model is applied to an external dataset. The external validation ensures predictability and applicability of the developed QSAR model for the prediction of untested molecules [34]. For that reason, the QSAR model was also externally validated by comparing the predicted and observed activities of the external test set of compounds with IC_50_ values measured in our laboratory (compounds in Fig. 1), which were not used in the model development. The IC_50_ values for these compounds were experimentally determined as described in the methods Sect. 2.2. It must be noted here that it is expected to observe interlaboratory variations as the training set compounds and test set compounds have been measured in two different laboratories using different techniques and equipment. Table 4 shows the observed and predicted pIC_50_ values for the external compounds. Despite the laboratory variations, the predicted pIC_50_ values correlate very well with the observed values as seen in Fig. 7. On the other hand, the predicted pIC_50_ values show large absolute error for some of these compounds. A closer comparison of the actual and predicted IC_50_ values in Table 4 shows that for extremely potent inhibitors (ascofuranone, ascochlorin, colletochlorin B), the actual IC_50_ of ~ 7 nM has been predicted to be at 0.2–0.7 range, amounting to a tenfold prediction error (logarithmic absolute error ~ 1). On the other end of the spectrum, propyl gallate is a weak inhibitor with an IC_50_ of 1,400 nM, predicted to be much weaker at 50,441 nM IC_50_ value. This indicates that the QSAR is valuable in predicting the rank order of compound’s inhibitory potency, but it may not be accurate in predicting the exact pIC_50_ values.

**Table 4:** Compounds used as external validation set, observed and predicted pIC_50_ values and absolute error

Compounds	pIC_50_ Exp	pIC_50_ Pred	Abs. error
Ascofuranone	8.131	9.262	1.131
Ascochlorin	8.131	9.183	1.052
Colletochlorin B	8.125	9.637	1.512
Colletochlorin D	7.481	9.009	1.528
SHAM	5.222	4.884	0.338
Propyl gallate	5.853	4.296	1.558
Octyl gallate	6.638	6.340	0.298
Ferulenol	7.275	7.010	0.266

## Discussion

Crystallisation of the trypanosomal alternative oxidase (TAO) has provided revealing insights into its α-helical structure and its attachment to the membrane [21]. Additionally, co-crystallization of TAO with a number of ascofuranone derivatives has provided considerable structural details with respect to the active-site and nature of the substrate/inhibitor-binding site. Such insights can lead to the generation of new AOX specific inhibitors. Ascofuranone (AF), for instance, is a naturally occurring compound isolated from *Ascochyta viciae*, which has been demonstrated to be a highly potent AOX inhibitor and to have trypanocidal activity both in vitro [19] and in vivo [20]. Despite its high potency against TAO, survival of mice following AF treatment showed that ascofuranone is not an effective inhibitor of the mammalian cytochrome *bc*_*1*_ complex, which makes it a promising clinical drug candidate. On the other hand, ascochlorin, which is a potent inhibitor of the AOX activity in both *Trypanosoma brucei* [17] and *Trypanosoma vivax* [35], is also a potent inhibitor of the cytochrome *bc*_*1*_ complex [37], making this compound unsafe clinically. SHAM and the gallates have generally been considered, and indeed used, as inhibitors to indicate the presence and potential contribution of AOX to the overall respiratory rate in isolated mitochondria, cells and tissues. However, outside of their use with isolated mitochondria, such inhibitors are not totally specific for AOX. For instance, it is well-documented that SHAM is able to inhibit other redox enzymes apart from AOX [36].

Knowledge on the nature of the substrate and inhibitor-binding site and catalytic mechanism of the enzyme is fundamental to a more rational design of highly targeted AOX inhibitors. The aim of this study was to explore molecular properties required for inhibition of AOX using computational modelling techniques, which can provide assistance for the future design of further AOX specific inhibitors. To achieve this, inhibitory activities of a series of ascofuranone derivatives towards AOX reported by Saimoto et al*. *[17] were used for molecular docking purposes and QSAR, and a series of molecularly diverse compounds (Fig. 1) were used to assess the validity of these computational techniques in the prediction of AOX inhibition.

Docking results and following PLIF analyses confirmed that the three most abundant residues involved in binding to inhibitors (AF derivatives in the training set) are Leu 122, Arg 118 and Thr 219 (Fig. 4). The significance of these key residues were further validated by docking of the test set compounds to the wild and mutated receptors, where mutations of L122A, R118A, T219A all caused a significant reduction in affinity, especially of the most potent inhibitors. The most crucial of these residues, Arg 118 and Thr 219, form hydrogen bonds with the hydrogen bonding functional groups on the meta position of the aromatic ring of the inhibitors, establishing a strong interaction between the compounds and the enzyme (see Fig. 5 for an illustration of these hydrogen bondings). The importance of this interaction was further confirmed by the QSAR analyses, which showed that the most important factor to control pIC_50_ values of the inhibitors is the presence of a hydrogen bond acceptor group on the meta position of the aromatic ring of the compounds. This is often a formyl group in the compounds tested here.

The QSAR analysis also highlighted the importance of acidity of the phenolic hydroxyl groups in the compounds, in addition to the impact of hydrogen bonding acceptor group on the meta position of the aromatic ring of ascofuranone derivatives, as discussed above. Moreover, a larger hydrocarbon linker group of the compounds with fewer electronegative heteroatoms lead to higher inhibitory potency towards AOX.

The QSAR model produced here was successfully validated by internal validation and external set of compounds, and the risk of chance correlation was also investigated. The external set consisted of a series of classic and novel AOX inhibitors with the pIC_50_ values measured in our laboratory. The QSAR model was able to predict the rank order of the pIC_50_ values of these diverse set of compounds, and a good estimate of potent (nM IC_50_ values) and weak (μM IC_50_ values), although the exact pIC_50_ values could not be predicted with a reasonable accuracy. This was probably due to the differences of experimental procedures used in our laboratory and those used by Saimoto et al*.* for the measurement of pIC_50_ values of the external compounds and the training set coumpounds, respectively. Moreover, within the external set of compounds, 3 have very similar nanomolar IC_50_ values (7.4, 7.4 and 7.5 nM), so the small difference in their IC_50_ values is unlikely to be significant considering the experimental error of the biological measurements (see Fig. 1). At the next level of potency, there are two compounds in the external set (Table 4) with IC_50_ values of 33 and 53 nM (colletochlorin D and ferulenol); these have been correctly identified to have higher IC_50_ values (lower pIC_50_s) than the aforementioned 3 compounds as seen in the figure. Lastly, within the weaker three compounds, propyl gallate has been mispredicted to be much weaker than it is, with predicted IC_50_ of ~ 50 μM, while the actual experimental value is only 1.4 μM. Despite this error, in practice, even this prediction can be useful to identify this compound as a weak inhibitor. Considering the diversity of these external set of compounds with respect to the training set, in terms of the molecular descriptors in the QSAR equation, values for the external set are within the range of the training set values, except for *PEOE_VSA_FPPOS* (see Supplementary data, Figure S3 for the histograms). The last three compounds have slightly higher *PEOE_VSA_FPPOS* than the data range in training set.

The AOX represents a promising target to address the threat posed by multiple human pathogenic organisms and numerous fungi of agronomic importance, particularly after the emergence of fungicide-resistant strains of phytopathogenic fungi [37, 38]. Hence, we conclude that the data presented here may prove to be useful tools for future design and development of novel and specific AOX inhibitors.

## Electronic supplementary material

Below is the link to the electronic supplementary material.

Supplementary file1 (DOCX 5085 KB)
