# Supplementary material for: QSAR and molecular docking for the search of AOX inhibitors: a rational drug discovery approach
Source: J Comput Aided Mol Des. 2020 Dec 8;35(2):245–60. doi: 10.1007/s10822-020-00360-8 (PMC7904559; doi:10.1007/s10822-020-00360-8)
Supplement: Supplementary file 1 — Supplementary file1 (DOCX 5085 KB) [file 10822_2020_360_MOESM1_ESM.docx]

# Supplementary data

**Table S1** Compounds used to develop the model, selected descriptors, experimental pIC_50_ values, calculated pIC_50_ values and absolute error. *LOO-cross validation absolute error values, ** 5-fold cross validation (leave-some-out) absolute error values.

| **Compounds** | **MetaH-bond acceptor** | **Neutral form** | **PEOE_VSA_FPPOS** | **Petit jean** | **pIC_50_ Exp.** | **pIC_50_ Calc.** | **Absolute error** | **Absolute error *** | **Absolute error **** |
| --- | --- | --- | --- | --- | --- | --- | --- | --- | --- |
| **1** | 1 | 0.07 | 0.076 | 0.500 | 9.398 | 9.338 | 0.060 | 0.065 | 0.250 |
| **2** | 1 | 0.07 | 0.087 | 0.462 | 8.921 | 8.471 | 0.450 | 0.479 | 0.398 |
| **3** | 1 | 0.07 | 0.085 | 0.467 | 8.854 | 8.603 | 0.251 | 0.264 | 0.058 |
| **4** | 1 | 0.1 | 0.048 | 0.467 | 9.523 | 9.196 | 0.327 | 0.359 | 0.647 |
| **5** | 1 | 0.04 | 0.052 | 0.467 | 9.000 | 9.264 | 0.264 | 0.288 | 0.042 |
| **6** | 1 | 0.1 | 0.052 | 0.467 | 9.301 | 9.116 | 0.185 | 0.201 | 0.985 |
| **7a** | 1 | 0.05 | 0.091 | 0.429 | 7.000 | 7.869 | 0.869 | 1.122 | 0.066 |
| **7b** | 1 | 0.04 | 0.079 | 0.444 | 8.420 | 8.390 | 0.030 | 0.034 | 0.265 |
| **7c** | 1 | 0.04 | 0.070 | 0.455 | 9.155 | 8.733 | 0.422 | 0.457 | 0.690 |
| **7d** | 1 | 0.04 | 0.063 | 0.462 | 9.420 | 8.988 | 0.433 | 0.464 | 0.318 |
| **7e** | 1 | 0.04 | 0.060 | 0.500 | 9.420 | 9.718 | 0.298 | 0.332 | 1.908 |
| **7f** | 1 | 0.04 | 0.054 | 0.500 | 9.347 | 9.815 | 0.468 | 0.529 | 1.359 |
| **8** | 1 | 0.1 | 0.071 | 0.474 | 9.301 | 8.894 | 0.407 | 0.422 | 1.122 |
| **9** | 1 | 0.1 | 0.075 | 0.500 | 9.523 | 9.292 | 0.231 | 0.250 | 0.508 |
| **10** | 1 | 0.1 | 0.127 | 0.500 | 9.495 | 8.350 | 1.145 | 1.535 | 0.065 |
| **11** | 1 | 0.1 | 0.096 | 0.462 | 8.222 | 8.242 | 0.020 | 0.022 | 0.184 |
| **12** | 1 | 0 | 0.139 | 0.462 | 7.398 | 7.713 | 0.316 | 0.442 | 0.029 |
| **13** | 1 | 0 | 0.115 | 0.471 | 8.377 | 8.306 | 0.071 | 0.081 | 0.299 |
| **14** | 1 | 0.17 | 0.046 | 0.467 | 8.602 | 9.042 | 0.440 | 0.483 | 1.137 |
| **15** | 1 | 0.17 | 0.074 | 0.500 | 9.155 | 9.135 | 0.020 | 0.022 | 0.078 |
| **16** | 1 | 0.17 | 0.057 | 0.500 | 9.824 | 9.436 | 0.388 | 0.422 | 0.401 |
| **17** | 1 | 0.83 | 0.070 | 0.500 | 7.553 | 7.540 | 0.013 | 0.019 | 0.393 |
| **18** | 1 | 0.24 | 0.094 | 0.462 | 7.347 | 7.924 | 0.578 | 0.637 | 0.307 |
| **19** | 0 | 0.77 | 0.063 | 0.462 | 5.000 | 4.881 | 0.119 | 0.301 | 0.393 |
| **20** | 1 | 0.01 | 0.081 | 0.462 | 9.347 | 8.731 | 0.616 | 0.654 | 0.164 |
| **21** | 1 | 0.07 | 0.082 | 0.500 | 8.222 | 9.243 | 1.021 | 1.116 | 0.726 |
| **22** | 1 | 0.23 | 0.079 | 0.500 | 7.000 | 8.890 | 1.890 | 2.033 | 0.050 |
| **23** | 0 | 0.01 | 0.062 | 0.462 | 6.699 | 6.818 | 0.119 | 0.301 | 0.460 |
| **24** | 1 | 0.07 | 0.063 | 0.500 | 10.222 | 9.586 | 0.636 | 0.699 | 0.332 |
| **25** | 1 | 0.06 | 0.050 | 0.467 | 9.347 | 9.250 | 0.097 | 0.106 | 1.568 |
| **26** | 1 | 0.06 | 0.063 | 0.500 | 9.638 | 9.606 | 0.032 | 0.035 | 0.160 |
| **27** | 1 | 0.63 | 0.063 | 0.500 | 9.301 | 8.165 | 1.136 | 1.385 | 0.697 |
| **28** | 1 | 0.58 | 0.067 | 0.500 | 7.420 | 8.228 | 0.808 | 0.956 | 0.018 |
| **29** | 1 | 0.6 | 0.023 | 0.467 | 8.398 | 8.378 | 0.020 | 0.029 | 0.422 |
|  |  |  |  |  |  |  |  |  |  |
| **MAE** |  |  |  |  |  |  | 0.417 | 0.487 | 0.485 |

**Figure S**1. Y-randomisation plot between r (Pearson correlation coefficient between pIC50 and the randomised IC50 vectors) and R^2^ or Q^2^ of the real and fake QSAR models obtained by stepwise regression analysis.

**Figure S2**. Y-randomisation plot between r and R^2^ or Q^2^, for the QSAR obtained from stepwise regression analysis following pre-processed feature selection using GeneticSearch with features evaluated using WrapperSubsetEval with Random Forest.


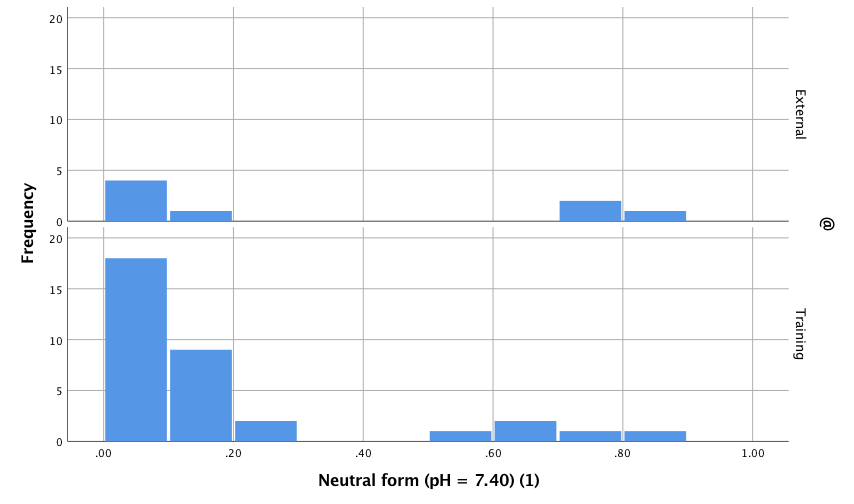

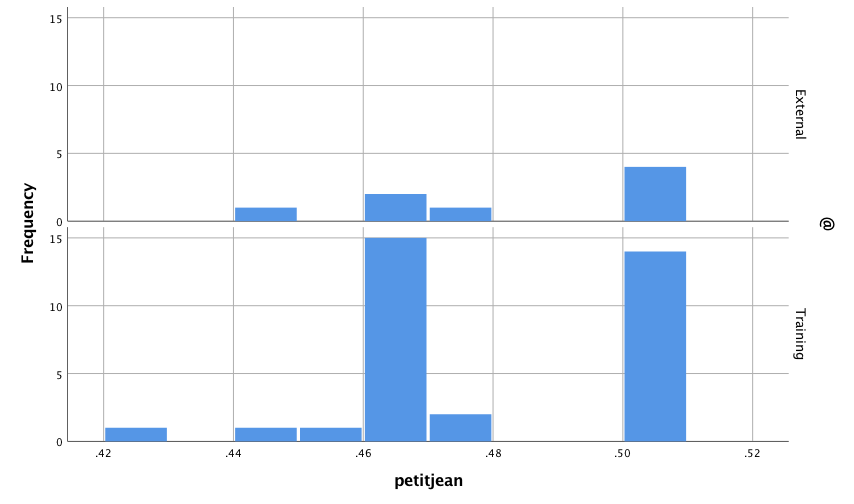

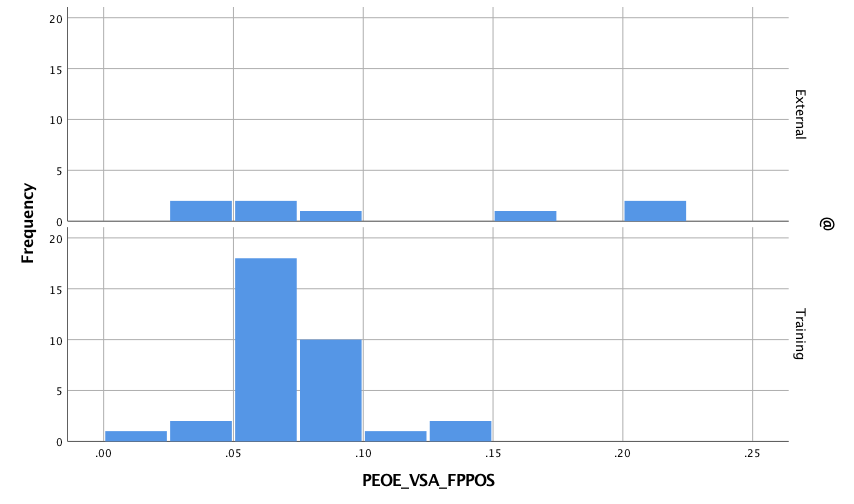

Figure S3. Histogram of the molecular descriptors used in the QSAR equation for training and test set compounds.
